# Supplementary material for: Enhancer of Zeste Homolog 2 (EZH2) Contributes to Rod Photoreceptor Death Process in Several Forms of Retinal Degeneration and Its Activity Can Serve as a Biomarker for Therapy Efficacy
Source: Int J Mol Sci. 2021 Aug 28;22(17):9331. doi: 10.3390/ijms22179331 (PMC8430630; doi:10.3390/ijms22179331)
Supplement: Supplementary file 1 [file ijms-22-09331-s001.zip › Supplemental_Figures_corrected.pdf]

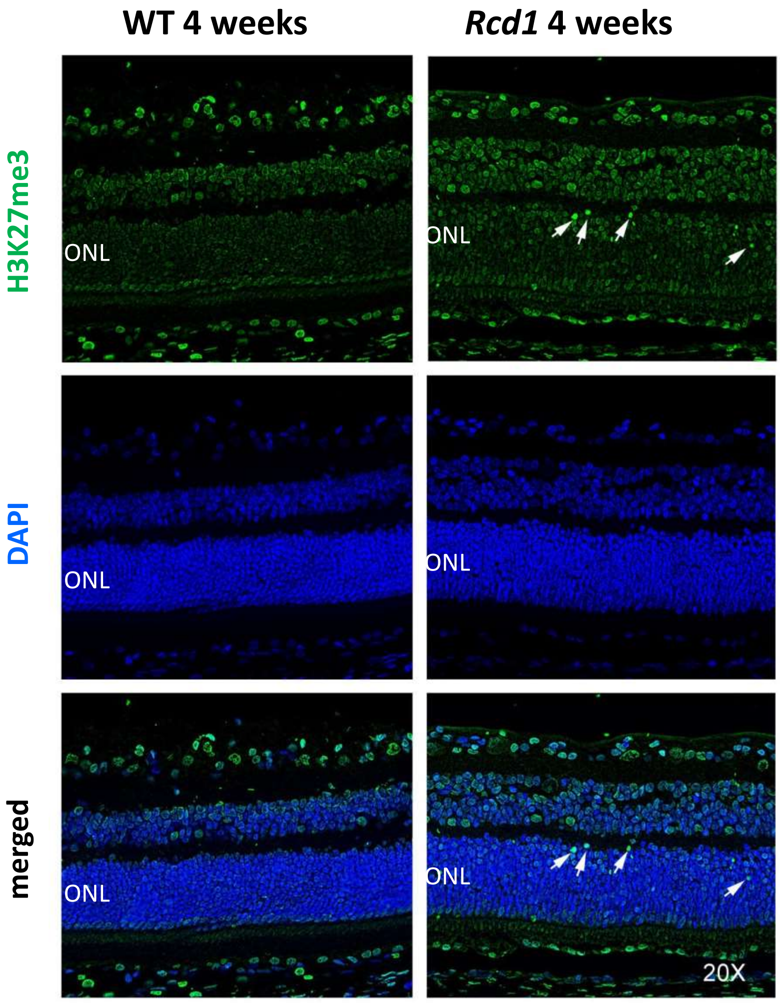

Figure S1: Inherited retinal degeneration in the *rcd1* dog is also characterized by H3K27me3 accumulation in the ONL. No accumulation of H3K27me3 mark was detected in the WT retina, whereas cell nuclei in the ONL of the *rcd1* dog contain high level of H3k27me3 (arrows). Note that the H3K27me3 accumulation is already present at 4 weeks of age which is 1 week after the onset of rod loss in this model. ONL = Outer Nuclear Layer

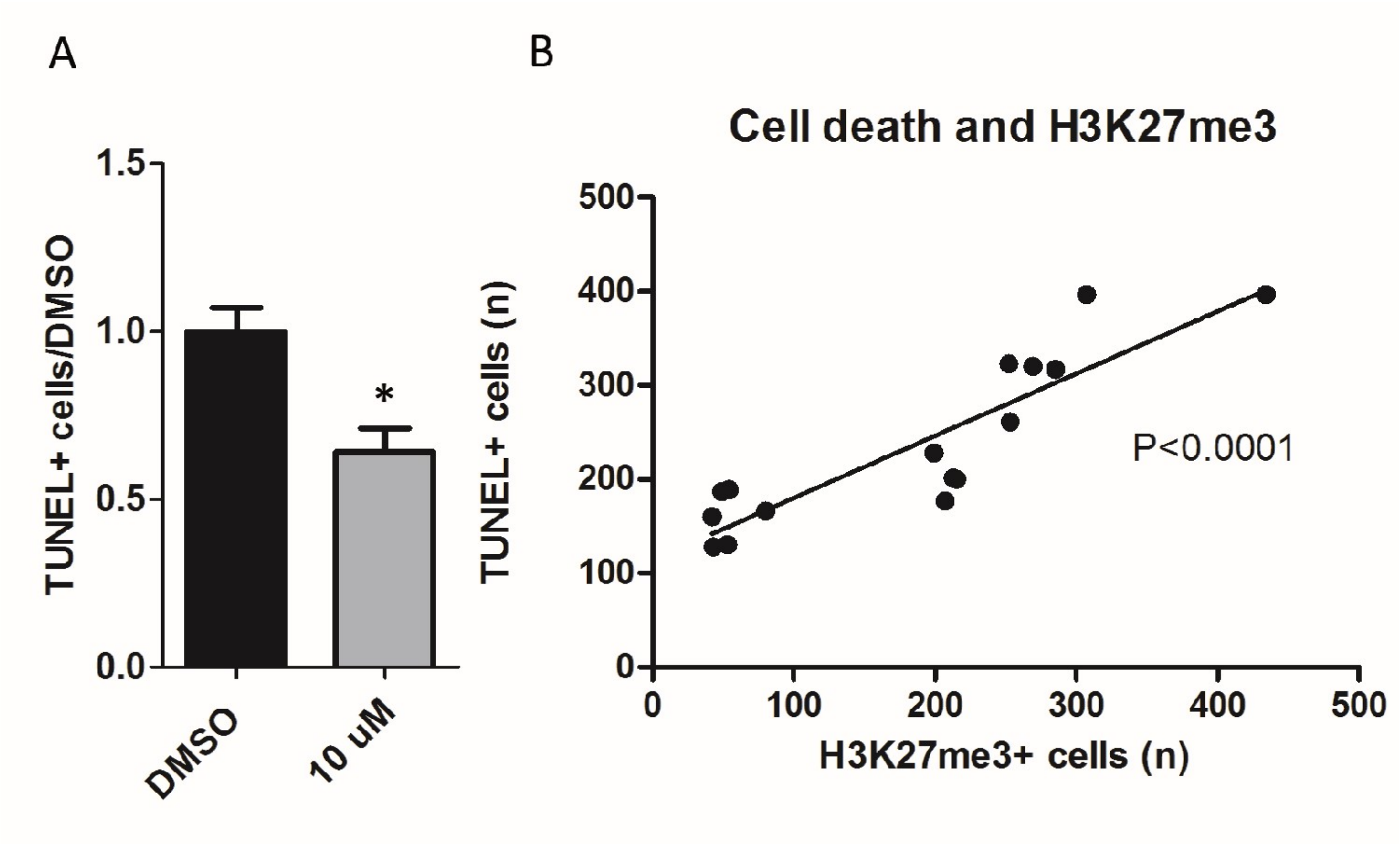

Figure S2: EPZ6438 treatment in the *Rd1* eye. (a). EPZ6438 injected at PN8-PN9 in *Rd1* retina reduced the number of TUNEL-positive cells. The mice were injected with 10 μM of EPZ6438 and analyzed at PN12. Statistical analysis: Mann Whitney test, n = 4, p = 0.029 (*). (b) All *Rd1* retina used in this study including non-injected retina, retina injected with DMSO, 5 or 10 μM of EPZ6438, with injection leakage or not, were analyzed for the potential relation between cell death and H3K27me3^{h}-positive cell number. The Pearson correlation analysis clearly shows a strong relationship between decreased level of H3K27me3^{h} and cell survival (P<0.0001).
